# Cortical source localization of sleep-stage specific oscillatory activity

**DOI:** 10.1038/s41598-020-63933-5

**Published:** 2020-04-24

**Authors:** Arianna Brancaccio, Davide Tabarelli, Marco Bigica, Daniel Baldauf

**Affiliations:** Center for Mind/Brain Sciences – CIMeC, University of Trento, Trento, Italy

**Keywords:** Non-REM sleep, Sleep

## Abstract

The oscillatory features of non-REM sleep states have been a subject of intense research over many decades. However, a systematic spatial characterization of the spectral features of cortical activity in each sleep state is not available yet. Here, we used magnetoencephalography (MEG) and electroencephalography (EEG) recordings during night sleep. We performed source reconstruction based on the individual subject’s anatomical magnetic resonance imaging (MRI) scans and spectral analysis on each non-REM sleep epoch in eight standard frequency bands, spanning the complete spectrum, and computed cortical source reconstructions of the spectral contrasts between each sleep state in comparison to the resting wakefulness. Despite not distinguishing periods of high and low activity within each sleep stage, our results provide new information about relative overall spectral changes in the non-REM sleep stages.

## Introduction

Brain activity both during wakefulness and sleep is characterized by fluctuations in neuronal responses and rhythmic activation at various time scales. Sleep occurs periodically following a rather strict circadian rhythm and is itself a highly dynamic event, characterized by re-occurring and alternating phases of circa 80–120 minutes each, during which different polysomnographic events can be recorded^1^. In each sleep stage, the brain is characterized by specific patterns of oscillatory activity. These oscillatory patterns of the sleeping brain have been described mostly using EEG. Our aim is to exploit the higher localization accuracy of magnetoencephalography (MEG) to shed new light on the spatial distribution of oscillatory features in non-REM sleep stages.

The first light sleep stage (N1) is a state of drowsiness and of early loss of consciousness, physiologically characterized by a decreasing low voltage EEG frequency (2–7 Hz)^2^. The following, second sleep stage (N2) is characterized by the occurrence of sleep spindles and K-complexes in the EEG signal. Spindles are rhythmic bursts of EEG activity that oscillate at a frequency between 12–15 Hz (classically named as sigma band). They have a waxing and a waning component and last for about 1 sec at a time^3,4^. K-complexes are variable patterns of sudden bursts consisting mostly of a high voltage diphasic slow wave, especially in N2. Their brief negative peak in the EEG seems to be a signature of neuronal hyperpolarization, while its initial positive component depends on excitation of neurons. Often K-complexes co-occur with sleep spindles or may even trigger them^5^. They are either spontaneous or occur in response to sudden sensory stimuli^6^. As sleep deepens, subjects enter a third sleep stage, N3. This is characterized by a reduction of sleep spindles and by the emergence of low-frequency, high-amplitude fluctuations (delta waves)^7,8^. In the past, some studies further distinguished between N3 and another N4 state, based on some arbitrary percentage of slow wave oscillations (e.g., >20% and >50%, respectively), but for the purpose of this study we consider them together. These three described stages (N1–3) follow each other in a systematic succession that goes from light to deep sleep and then back to light sleep. Since N1 is more a transition stage between wakefulness and sleep (often sporadic and linked to random sleep events, e.g. following arousals or awakenings), it does not necessarily re-occur consistently after N3 in subsequent cycles, i.e., N2 and N3 may systematically alternate. Only after several of those cycles through sleep stages N1–3 subjects may enter yet a further sleep stage, which is characterized by rapid eye movements and high-frequency, low amplitude EEG activity, similar to the EEG observed during restful wakefulness (REM)^8,9^. These latter REM sleep phases are more consistently found during the second half of the night.

In the past, both MEG and EEG have been used to investigate specific sleep features like cross-cortical connectivity^10–12^, the neural origin of sleep spindles^3,13–20^ and perceptual or cognitive changes during sleep^21–27^.

Among the existing literature, studies focusing specifically on spectral features of sleep MEG recorded brain activity are mostly related to specific events (sleep spindles, eye movements during REM, slow wave fluctuations^3,11,19,28–30^ and/or to memory consolidation and perceptual learning^13,29,31^.

For example, Klinzing *et al*.^19^ investigated with MEG, in the spectral domain, the relationship between sleep spindles and slow wave oscillations (<1 Hz), as well as the spatial distribution of spindle spectral profiles over the cortex. In a set of eleven subjects, they found a phase locking of fast spindle activity (12–15 Hz) to the depolarizing up-state of slow waves and of slow spindles activity (9–12 Hz) to the up-to-down transition of the slow oscillation. In addition, by reconstructing cortical spectral power using DICS^32^, they observed a less distinct anterior–posterior separation of fast and slow spindles with respect to what usually observed in EEG. Spindle events have also been related to power changes in gamma activity in the context of memory consolidation during sleep. Ayoub and colleagues^3^ investigated how the gamma oscillatory activity (>30 Hz), detected by MEG during sleep and classically related to memory consolidation, is related to sleep spindles, as detected by EEG recordings. From a sample of seven participants, they showed that in prefrontal and occipital areas the MEG spindle power (12.5–15.5 Hz) was greater and the modulations in gamma power occurred more strongly. Also, cross-frequency coherence analyses confirmed that gamma band and the spindle rhythm are strongly phase-coupled. However, the analysis has been performed without any cortical reconstruction and only at the sensor level. From another perspective, ultraslow power modulations of oscillatory activity during sleep has been linked to memory consolidation and post learning reactivation. In a set of eight participants, Piantoni *et al*.^29^ found that a long-range pattern of synchronization of ultra-slow power fluctuations is elicited by a visuo-motor task in the beta band during wakefulness and that the parieto-occipital component of this pattern reappears in the delta band during N2 sleep stage. Ultra-slow power modulation of spontaneous activity during wakeful rest and sleep has been investigated also *per se* by Liu *et al*.^11^ On a sample of seven healthy subjects and analysing data only at the sensor level, they found the power spectra of all classical frequency bands (delta, theta, alpha, beta and gamma) being spontaneously modulated in an ultra-slow regime (<0.1 Hz) and the modulations were noticeably stronger in light sleep than in awake. Moreover, coherence on band limited power envelopes, indicates the ultraslow modulation tends to synchronize over a long spatial distance, in particular between homologous regions in opposite hemispheres. Power spectrum changes at the cortical level in sigma and delta band during sleep have also been investigated with respect to perceptual learning. Bang *et al*.^13^ addressed how slow/fast sigma and delta oscillatory activity change in early visual areas during sleep after a visual perceptual learning task. On a set of 15 participants, they found trained regions of visual cortices exhibiting a performance gain correlated power increase in slow sigma band (11.5–12.5 Hz) in N2 but not during slow waves sleep states. No fast sigma or delta waves power changes has been found with respect to perceptual learning. Tamaki *et al*.^31^ investigated cortical delta and fast sigma activity in relation to consolidation of pre-learned motor-sequences. In a set of ten participants, after a finger-tapping motor sequence learning, they found spontaneous delta and sigma fast- oscillations significantly increased in the supplementary motor area (SMA) during post-training compared with pre-training sleep, showing significant and high correlation with the performance increase. Among the literature, other studies partially addressing the spectral features of MEG brain activity are linked to rapid eye movements and the First Night Effect (FNE). Corsi-Cabrera and colleagues^28^ investigated how gamma band (32–48 Hz) power changed during REM sleep before/during and in absence of rapid eye movements. They collected EEG data for ten participants and reanalysed MEG data of four subjects, collected from another study (Ioannides *et al*.^33^). Even without any cortical localization, they found both EEG and MEG gamma activity being higher directly before/during eye movement in REM sleep. Finally, Tamaki *et al*.^30^ addressed the impact of the environment (First Night Effect - FNE) on slow wave spontaneous oscillatory activity (1–4 Hz) in the visual areas. On a total of ten subjects, they reconstructed brain activity from MEG data during two consecutive sleep sessions. They found the strength of slow waves oscillations was reduced in the first sleep session with respect to the second one, especially in ventral visual areas.

Only a few studies directly tackle the issue of how the spectral profile of spontaneous oscillatory activity in the brain, as seen with MEG, changes systematically among sleep stages. On a set of four subjects, Manshanden *et al*.^34^ investigated the spatial location of sources for spindle activity in N2 sleep and alpha and mu rhythms during wakefulness. Using a multiple equivalent current dipole model to localize cortical activity from MEG and EEG recordings, they found spindles, alpha and mu rhythms generators being overlapped but distinct; in particular, distributed over the centro-parietal region, around the occipito-parietal sulcus and around the central sulcus, respectively. However, they did not directly compare the reconstructed cortical locations for different rhythms between sleep and wakefulness. In another study, Simon and colleagues^35^ addressed the evolution of power spectral density of MEG signal during the evolution from wakefulness to light sleep. They observed a reduction in alpha as three subjects entered N1 and some increase in slower waves. As for the deep sleep, they only used data from one subject from which they registered an increase of slow oscillations (in their study: 0.3–0.7 Hz) especially at the level of the temporal lobe. The spectral features are addressed mostly at the sensor level, whereas an equivalent dipole model has been used to address only a few characteristics of sleep. No anatomical constrain (neither individual MRI or standard anatomies) were used in the source reconstruction. Moreover, only one out of three subjects entered deep sleep, given that they could not sleep for more than 30 minutes consecutively because they were constrained in a sitting position in the scanner. Finally, data were filtered at 60 Hz, thus no gamma activity could be investigated.

Probably the most relevant studies addressing general spectral features of sleep brain activity are those of Ioannides *et al*.^36^ and Ioannides *et al*.^37^.

Ioannides *et al*.^36^ focused their attention on *core* periods of sleep activity, namely relatively quiescent periods without grapho-elements or high activity. They recorded MEG during whole night sleep, after an acclimatization night, on a set of four subject. One of the subjects was sleep deprived. They mostly tried to detect, using individual MRI assisted Magnetic Field Tomography, focal generators of gamma activity (25–90 Hz) as indicator of cortical activation. Moreover, they report results also for spectral features in other classical frequency bands (delta 1–3 Hz, theta 5–7 Hz, alpha 8–13 Hz and beta 15–25 Hz), but only for selected regions of interest. These were first identified by exploiting in which areas power spectral changes were significantly different when comparing each sleep stage with eyes closed wakefulness in broad band activity (1–200 Hz) and gamma activity for all subjects. Power spectral changes in the ROIs, which turned out to be significant in the previous analysis, were then investigated, by comparing each stage with eyes closed wakefulness. The total amount of core data analyzed per subject and per sleep stage and wakefulness consisted in eight four seconds periods (best case). As regards gamma activity, they found a gamma power increase in precuneus during light sleep (N1 and N2) and in left Dorsal Medio Pre Frontal Cortex (L-DMPFC) in deep sleep (N3 and N4 and REM). Gamma activity in L-DMPFC was higher and spatially more extended in REM than in all other stages, with respect to wakefulness, even if reduced in parietal cortex and Posterior Cingulate Cortex. As for the classical frequency bands, in the areas identified by contrasting sleep stages and awake for broad band and gamma oscillatory activity, they highlight that power spectral density below alpha was higher in all sleep stages than in awake, that, as expected, alpha activity peaks more in awake for posterior regions but also in L-DMPFC and that, in REM, a reduced peak of alpha activity re-appears in posterior ROIs. Even if mostly focused on gamma, this study provides at least partial spatial information about power spectral changes in other classical bands for periods of *quiescent* sleep activity. However, the small sample size and the inhomogeneity of the sample (only four subjects, one sleep deprived) suggest that further investigations are required.

In Ioannides *et al*.^37^, the authors identified regional spectral changes at the sleep onset and during light sleep. Their goal was to provide a comprehensive description of changes from awake to light sleep. For this reason, they restricted the analysis to eyes closed wakefulness data, N1 and N2. From the same set of four subjects analysed in Ioannides *et al*.^36^, they reconstructed brain activity using individual MRI assisted Magnetic Field Tomography and investigated both the relative power spectral changes in *core* quiescent periods and in activity pre/during characteristic events (spindles and K-complexes). As for the first analysis, which is the more relevant to our study, the authors used five frequency bands (delta 3.2–6.4 Hz, theta 4.8–8 Hz, alpha 8–11.2 Hz, low-sigma 9.8–12.8 Hz and high-sigma 12.8–16 Hz), detecting power changes of N1 and N2 with respect to eyes closed wakefulness and between N1 and N2. They found a progressive increase in frontal delta and theta activity as subject progress from awake to N1 and N2, and a correspondent reduction in alpha and higher frequencies in posterior parietal cortex. Direct comparison of N2 with N1 showed modest fronto-parietal increases in delta and theta power, together with focal frontal and anterior-cingulate increases in the alpha and low-sigma band.

Taken together, Ioannides *et al*.^36^ and Ioannides *et al*.^37^ are, to our knowledge, the best available characterizations of how MEG reconstructed power spectral density changes across sleep stages in healthy humans. While robust analysis and programmatic exclusion of high activity and grapho-elements make the aforementioned results highly significant, they suffer of very small sample size (four subjects) and some sample inhomogeneity (three subjects not deprived, one deprived). As also the authors suggest, further studies with more subjects are needed in order to generalize results^37^.

Therefore, a comprehensive spatial characterization of the spectral features of brain activity, as seen by MEG in each sleep stage, is still missing. Here, we tried to fill this gap by investigating, despite without distinguishing between periods of high/low activity, how the spectral power density in classical frequency bands changes from awake to non-REM sleep and localizing them in particular at the superficial cortical level. Specifically, we recorded MEG activity during sleep stages N1–3.

We combined the recorded data with each subject’s individual anatomical MRI, which allows for a source-localization of the oscillatory *cortical* signatures in the subjects’ brain (see the *Discussion* section about limitations in the source reconstruction we used). We were interested in investigating spectral features in the classical frequency bands: delta (1–4 Hz), theta (4–8 Hz), beta (14–30 Hz), alpha (individual alpha peaks), low gamma (30–60 Hz) and high gamma (60–100 Hz). In addition, we included relevant frequency bands, as slow waves (0.2–1.2 Hz) and sigma (12–15 Hz). For each subject, the amplitude spectral density (ASD) was computed separately for each stage. Amplitude spectral densities at the cortical level were then averaged over each subject’s sleep stage and then reduced to a set of 180 regions of interest (ROI), as the maximal ASD across all the vertices within a given ROI. We used a state-of-the-art parcellation of the human brain^38,39^. For each ROI we computed the difference in the amplitude spectral density (ASD) between each specific sleep stage (N1, N2, N3) and the awake resting state with eyes closed (Wake), respectively. A parametric dependent-samples t-test for each contrast was performed (p < 0.05, FDR corrected). Additionally, the same procedure was applied to all other contrasts between sleep stages (N2 vs N1; N3 vs N1; N3 vs N2).

## Results

We recorded in total 1305 min of sleep, i.e., on average 145 min per participant (range 55–264.3 min). From sleep stage classification by two independent human raters, we obtained a total of 206.2 min of wakefulness, 243.5 min of N1 sleep, 537.1 min of N2 sleep and 452.6 min of N3 sleep. On average we recorded 22.9 min (SEM = 5.1) of wakefulness, 27.1 min (SEM = 5.2) of N1, 59.7 min (SEM = 13.8) of N2, and 50.3 min (SEM = 8.4) of N3 per subject. The individual subjects’ sleep classifications are summarized in Table 1. Hypnograms can be inspected in Supplementary Fig. 1. As can be noticed, only three out of nine subjects reached REM sleep, thus showing at least one sleep cycle. We did not analyze REM stage since not all subjects reached this stage. These results have to be contextualized in the inevitable limitations of MEG sleep research, where having the head constrained and being not free to move might affect subjects’ sleep. We decided to include in the analysis all these subjects since all of them reached at least once all the non-REM sleep states, having at least five minutes of recording in each sleep stage. We considered this criterion in line with standard MEG research on awake resting state, which is usually based on a minimum of five minutes’ recordings^40–42^.

**Table 1:** Overview of the subjects’ sample with the respective amounts of sleep data recorded.

SUBJECT	GENDER	AGE	HANDEDNESS	SLEEP TIME (minutes)					
				WAKE	N1	N2	N3	REM	TOTAL
S1	M	24	right	23.9	18.5	42.4	26.2		87,1
S2	M	29	right	31.5	26.6	29.9	23		79,5
S3	F	31	right	12.7	9	14.2	53.4		76,6
S4	M	24	right	5.9	30.6	13.1	11.3		55
S5	M	25	right	13.5	45.1	123.3	63.8	18	250,2
S6	M	26	right	25	40	65.9	57.2		163,1
S7	F	25	right	37.5	50.4	123.8	55.1	35	264,3
S8	M	26	right	5	6.8	54.7	78.1	18.9	158,5
S9	F	23	right	51.2	16.5	69.8	84.6		170,9
**TOT**				206.2	243.5	537.1	452.7	71.9	1305
**AVERAGE**		25.8		22.9	27.1	59.7	50.3	24	145

As regards power spectra, in general, different sleep stages were characterized by well-known rhythmic patterns. Compared to the state of full wakefulness, sleep stage N2 showed an increase in low-frequency spectral magnitude in the delta and theta range, as well as a decrease in high-frequency bands (gamma range). In addition, N3 was characterized by strong slow oscillations below 1 Hz. N2 and N3 both showed characteristic spectral activity compatible with sleep sigma spectral range. Across sleep stages N1 to N3 we found a steady increase in slow oscillation activity, and a continuous decrease in high-frequency oscillations from light to deeper sleep stages (see Supplementary material for direct statistical comparisons).

In the following, we report detailed results for all the performed comparisons between sleep stages and awake. It has to be noticed that, given the intrinsic bias of the Minimum Norm Estimate solution, we do not emphasize source localization of power changes in deep structures, as they can be beyond the range of MNE.

### N1 vs Wake: decreased activity in the alpha band of visual cortices

**Figure 1:**
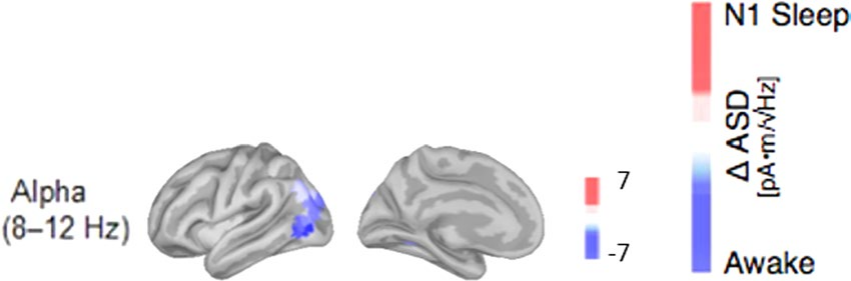
Cortical reconstructions of the neural sources for observed oscillatory patterns during N1 sleep in comparison to the awake resting state with closed eyes. Only in the alpha band (8–12 Hz) the contrast showed significant differences. Red correspond to amplitude increases relative to the awake state, blue represent decreases in spectral amplitude in the respective frequency band; all activity changes are thresholded at p < 0.05, FDR-corrected.

The comparison between N1 sleep stage and wakefulness was characterized by significant changes in the amplitude spectral density of the alpha-band. In comparison with the awake state with eyes closed, the ASD decreased significantly in visual areas of the occipital cortex (see Fig. 1 and Table 2).

**Table 2:** List of the brain regions that showed the strongest absolute changes in the spectral power between the N1 sleep and the awake stage (see Fig. 1, units are in pA*m/√Hz).

N1 - Wake	Roi	Δ Asd	p-value
**ALPHA**	V4t	−6.79	0.0003
	FST	−5.60	0.0003
	MST	−5.31	0.0002
	MT	−4.52	0.0001
	PGp	−4.21	0.0002
	V3CD	−4.12	0.0001
	V3A	−3.94	0.0003
	LO3	−3.79	0.0004
	V3B	−3.43	<0.0001
	IP0	−3.37	<0.0001

Brain regions are defined and labeled according to the brain parcellation by Glasser and colleagues^38^. Only the 10 areas with the strongest and significant changes in amplitude spectra are shown with their respective p-values.

Abbreviations used in Tables 2–4: 10pp: polar 10p, 13 l: area 13 l lateral to the orbital frontal cortex, 31pv: area 31p ventral in the posterior cingulate, 44: area 44 in the inferior frontal cortex, 46: area 46 in the dorso-lateral prefrontal cortex, 55b: area 55b in the premotor cortex, 6a: dorsal area 6 in the premotor cortex, 6r: rostral area 6, 6 v: ventral area 6 in the premotor cortex, 7am: medial area 7 A (in the superior parietal cortex), 8Ad: area 8Ad in the dorso-lateral prefrontal cortex, 8Av: area 8Av in the dorso-lateral prefrontal cortex, 8 C: area 8 C in the dorso-lateral prefrontal cortex, 9–46d: area 9–46d, a32pr: area anterior 32 prime in the paralimbic cortex, a47r anterior a47 area (in the inferior-frontal cortex), a9–46v: area a9–46dv, AVI: anterior insular, EC: ectorhinal cortex, FEF: frontal eye fields, FFC: fusiform face complex, FST: area FST in the MT + complex, H: hippocampus, i6–8: area i6–8 in the dorso-lateral prefrontal cortex, IFJa: anterior inferior-frontal junction, IFSp: posterior inferior-frontal sulcus, IP0: intra-parietal area 0, LO1: lateral-occipital area 1, LO2: lateral-occipital area 2, LO3: lateral-occipital area 3, MST: medial superior-temporal area, MT: middle temporal area, p10p: area posterior 10 P, PBelt: para-belt complex, PeEc: perirhinal ectorhinal cortex, PEF: premotor eye field, PGp: area PGp in the inferior parietal cortex, PH: area PH, PHT: area PHT, PHT: area PHT, PI: para-insular area, PIT: posterior inferio-temporal, ProS: pro-striate area, RSC: retro-splenial complex, STSdp: dorsal superior temporal sulcus (posterior), STSva: ventral anterior part of superior temporal sulcus, STSvp: ventral posterior part of superior temporal sulcus, TA2: area TA2 in the auditory association cortex, TE1a: anterior part of area TE1 in the middle temporal gyrus, TE1m: middle part of area TE1 in the middle temporal gyrus, TE1p: posterior part of area TE1 in the middle temporal gyrus, TE2a: anterior part of area TE2 in the Lateral temporal cortex, TE2p: posterior part of area TE2 in the Lateral temporal cortex, TGd: dorsal area TG in the temporal polar cortex, TGv: ventral area TG in the temporal polar cortex, TPOJ1: temporo-parieto occipital junction 1, V1: primary visual cortex, V2: second visual area, V3: third visual area, V3B: area V3B in the dorsal stream, V3CD: area V3CD in the MT + complex, between PGp and V4, V4: fourth visual area, V4t: area V4t in the MT + complex, V6A: anterior part of area V6 (between V6 and IPS1 in the parieto-occipital sulcus), V7: seventh visual area, V8: eighth visual area, VMV1: ventro-medial visual area 1, VMV3: ventro-medial visual area 3, VVC: ventral visual complex.

### N2 vs Wake: increased activity in low-frequencies (slow-wave, delta, theta, and sigma), combined with decreased activity of high-frequency bands

**Figure 2:**
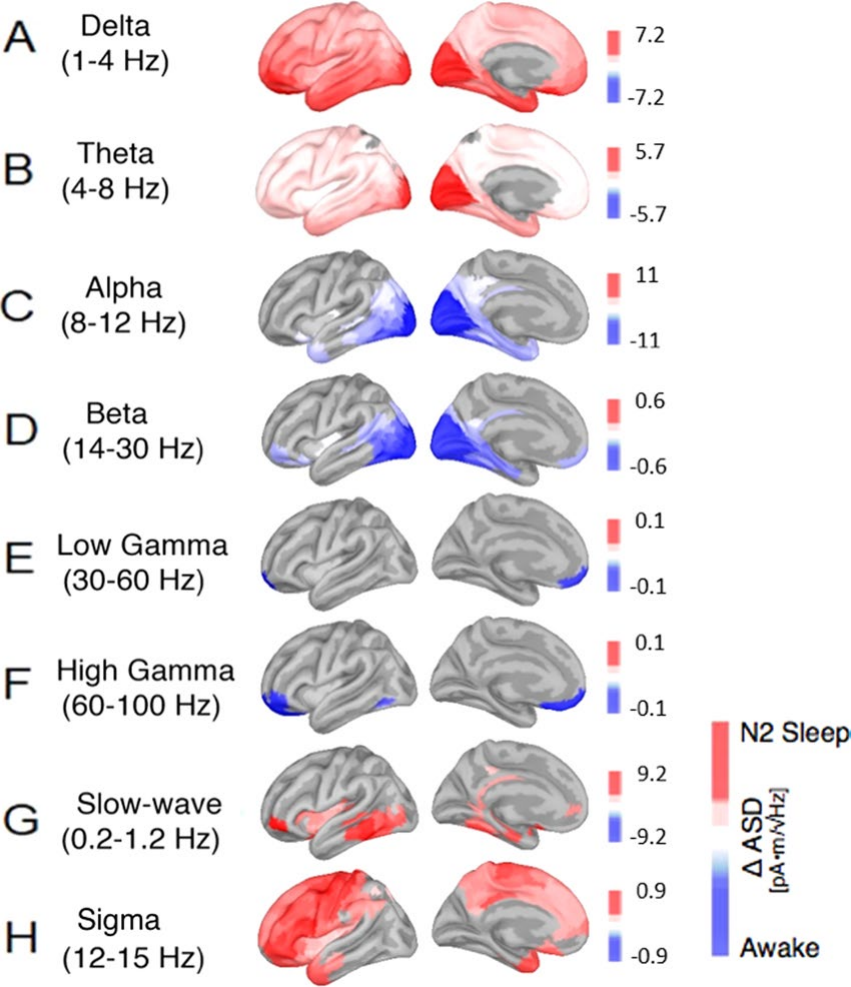
Cortical reconstructions of the neural sources for observed oscillatory patterns during N2 sleep in comparison to the awake resting state with closed eyes. Panels (A–H) show various general and sleep- specific frequency bands: (**A**) delta (1–4 Hz), (**B**) theta (4–8 Hz), (**C**) alpha (8–12 Hz), (**D**) beta (14–30 Hz), (**E**) low gamma (30–60 Hz), (**F**) high gamma (60–100 Hz), (**G**) slow wave oscillations (0.2–1.2 Hz) and (**H**) the sleep spindle spectrum (12–15 Hz). Red correspond to amplitude increases relative to the awake state, blue represent decreases in spectral amplitude in the respective frequency band; all activity changes are thresholded at p < 0.05, FDR-corrected.

In N2 sleep, increased activity in the delta band was mostly source-localized to the occipital cortex, inferior- and lateral-temporal cortex (IT) and frontal cortex (Fig. 2A and Table 3).

**Table 3:** List of the brain regions that showed the strongest absolute changes in the spectral power between the N2 sleep and the awake stage (see Fig. 2, units are in pA*m/√Hz). Abbreviations as in Table 2.

N2 - Wake	Roi	Δ Asd	p-value		Roi	Δ Asd	p-value
**DELTA**	OFC	7.15	<0.0001	**LOW GAMMA**	10pp	−0.14	0.0048
	V1	6.86	0.0005		10 v	−0.13	0.0032
	V2	6.62	0.0002				
	a47r	6.58	0.0003				
	V3	6.25	0.0002				
	PeEc	6.08	0.0001				
	TE2a	6.02	0.0001				
	47 l	5.99	0.0005				
	11 l	5.97	0.0002				
	PIT	5.95	0.0004				
**THETA**	V1	5.73	0.0120	**HIGH GAMMA**	10pp	−0.13	0.0011
	V2	5.28	0.0105		OFC	−0.12	0.0011
	V3	5.19	0.0144		10 v	−0.12	0.0005
	V4	4.05	0.0058		11 l	−0.12	0.0178
	PIT	3.48	0.0151		a47r	−0.11	0.0085
	V8	3.08	0.0022		PH	−0.09	0.0219
	FFC	3.05	0.0162				
	LO2	3.04	0.0072				
	PH	2.95	0.0086				
	V4t	2.94	0.0099				
**ALPHA**	V2	−11	0.0014	**SLOW OSCILLATIONS**	a47r	9.19	0.0195
	V4	−11	0.0047		TE1p	8.99	0.0066
	V3	−10	0.0052		pOFC	8.62	0.0219
	V1	−10	0.0044		TE2p	8.52	0.0052
	PIT	−10	0.0008		TE1m	8.04	0.0092
	V8	−9.31	0.0017		PH	7.98	0.0141
	FFC	−8.51	0.0015		FST	7.59	0.0042
	PH	−8.37	0.0006		VVC	7.57	0.0189
	V4t	−8.05	0.0003		LO2	7.45	0.0081
	LO2	−7.93	0.0006		STSdp	7.34	0.0004
**BETA**	PIT	−0.61	0.0072	**SIGMA**	FEF	0.93	0.0006
	LO2	−0.59	0.0069		6r	0.90	0.0002
	V8	−0.57	0.0042		44	0.88	0.0002
	PH	−0.56	0.0021		6d	0.86	0.0029
	V4	−0.56	0.0054		3b	0.85	0.0014
	V2	−0.55	0.0042		p47r	0.83	0.0059
	V4t	−0.54	0.0038		47 l	0.83	0.0041
	FFC	−0.54	0.0093		6a	0.82	0.0011
	V3	−0.53	0.0046		45	0.82	0.0012
	V1	−0.52	0.0162		PEF	0.82	0.0003

Also, the theta band increased in the contrast between N2 and the awake state and its main activation was localized to occipital cortex, here especially early visual areas V1, V2, V3 and V4 (see Fig. 2B and Table 3).

Compared to wakefulness, the alpha band shows a decrease at the level of occipital and temporal regions (see Fig. 2C). In the beta band, source localization showed again a decrease especially in occipital and frontal regions (see Fig. 2D). The reduction in low gamma (30–60 Hz) and high gamma (60–100 Hz) in N2 was mostly source localized in orbitofrontal and medial prefrontal areas (Fig. 2E,F).

The slow-wave spectrum (0.2–1.2 Hz), however exhibited significant increases especially in temporal and medial prefrontal areas (see Fig. 2G). Also, the power spectrum of sleep sigma activity (12–15 Hz) further increased, particularly in medial-frontal regions around the frontal eye fields (FEF), the dorso-lateral inferior-frontal and parietal cortex, the insula and the operculum (see Fig. 2H and Table 3).

### N3 vs Wake: systematic changes in low- and high-frequency bands and sigma band

**Figure 3:**
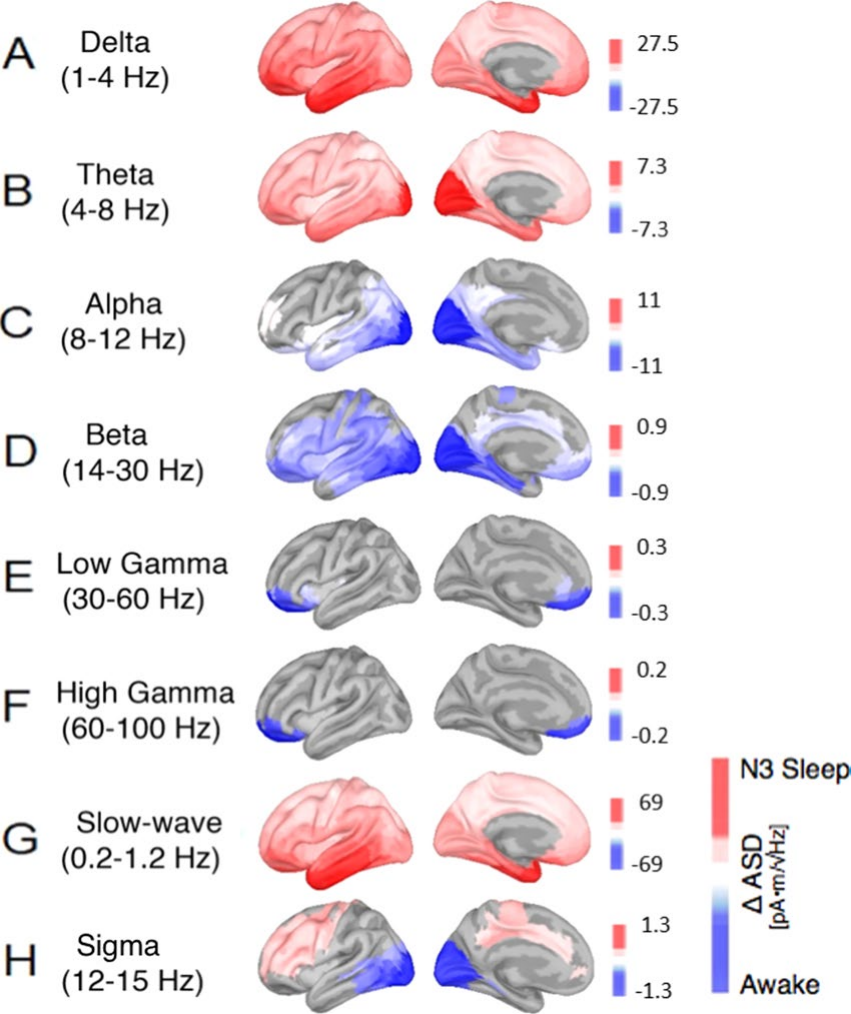
Cortical reconstructions of the neural sources for observed oscillatory patterns during N3 sleep in comparison to the awake resting state with closed eyes. Panels (A–H) show various general and sleep- specific frequency bands: (**A**) delta (1–4 Hz), (**B**) theta (4–8 Hz), (**C**) alpha (8–12 Hz), (**D**) beta (14–30 Hz), (**E**) low gamma (30–60 Hz), (**F**) high gamma (60–100 Hz), (**G**) slow wave oscillations (0.2–1.2 Hz) and (**H**) the sleep spindle spectrum (12–15 Hz). Red correspond to amplitude increases relative to the awake state, blue represent decreases in spectral amplitude in the respective frequency band; all activity changes are thresholded at p < 0.05, FDR-corrected.

Low-frequency activity, both in the delta and theta band, increased steadily from light to deeper sleep stages. In particular, delta band activity and slow-wave activity was much stronger in N3 compared to wakefulness, and source-reconstructions revealed that this was especially the case in fronto-temporal regions (see Fig. 3A,G and Table 4). Also, theta further increased and showed a specific cortical origin in visual areas of occipital cortex, particularly around visual areas V1, V2/3, and V4 (see Fig. 3B and Table 4).

**Table 4:** List of the brain regions that showed the strongest absolute changes in the spectral power between the N3 sleep and the awake stage (see Fig. 3, units are in pA*m/√Hz). Abbreviations as in Table 2.

N3 - Wake	Roi	Δ Asd	p-value		Roi	Δ Asd	p-value
**DELTA**	TE2a	27.45	<0.0001	**LOW GAMMA**	10pp	−0.28	0.0016
	TE1a	26.49	0.0001		OFC	−0.26	0.0093
	TGd	25.96	0.0001		10 v	−0.24	0.0080
	PeEc	25.89	<0.0001		11 l	−0.21	0.0029
	TE1m	25.33	0.0001		a47r	−0.19	0.0079
	TF	24.85	0.0001		13 l	−0.18	0.0185
	TGv	24.84	<0.0001		10r	−0.18	0.0017
	OFC	24.24	<0.0001		47 s	−0.16	0.0131
	a47r	24.01	0.0001		s32	−0.15	0.0201
	TE1p	23.34	<0.0001		AVl	−0.11	0.0185
**THETA**	V1	7.33	0.0012	**HIGH GAMMA**	10pp	−0.23	0.0027
	V3	7.24	0.0012		OFC	−0.21	0.0106
	V2	7.23	0.0005		10 v	−0.19	0.0247
	V4	6.01	0.0002		11 l	−0.19	0.0035
	PIT	5.27	0.0007		13 l	−0.18	0.0174
	V8	5.05	<0.0001		a47r	−0.17	0.0090
	PH	4.87	0.0001		47 s	−0.16	0.0128
	FFC	4.85	0.0004		10r	−0.13	0.0043
	TE2a	4.8	0.0001				
	PeEc	4.73	<0.0001				
**ALPHA**	V2	−11.03	0.0021	**SLOW OSCILLATIONS**	TE2a	68.63	<0.0001
	V4	−11.03	0.0062		TE1a	64.77	0.0001
	V3	−10.81	0.0057		PeEc	64.01	0.0001
	V1	−10.39	0.0056		TGd	63.76	0.0002
	PIT	−10.25	0.0013		TE1p	62.82	<0.0001
	V8	−9.37	0.0016		TE1m	62.32	0.0001
	FFC	−8.62	0.0018		TGv	61.87	0.0001
	PH	−8.57	0.0008		TF	60.82	0.0001
	V4t	−8.44	0.0003		TE2p	60.47	<0.0001
	LO2	−8.33	0.0007		STSvp	56.63	<0.0001
**BETA**	PIT	−0.94	0.0011	**SIGMA**	LO2	−1.26	0.0081
	LO2	−0.91	0.0007		PIT	−1.19	0.0082
	V1	−0.89	0.0020		V2	−1.16	0.0183
	V2	−0.89	0.0006		V4t	−1.15	0.0083
	V4	−0.85	0.0011		V4	−1.11	0.0210
	V4t	−0.83	0.0006		V3	−1.08	0.0208
	PH	−0.83	0.0004		FST	−1.06	0.0060
	V3	−0.82	0.0005		V1	−1.04	0.0274
	FST	−0.79	0.0004		PH	−1.01	0.0095
	FFC	−0.78	0.0021		MST	−0.99	0.0058

Higher frequencies in the alpha range (8–12 Hz) were significantly reduced in N3 compared to wakefulness, particularly in the occipital visual areas and temporal areas (see Fig. 3C). Also, activity in the beta frequency range (14–30 Hz) was significantly reduced in N3 compared to wakefulness, with cortical topography in frontal cortex, around motor-related areas, and occipito-temporal brain regions, particularly around cortical areas along the ventral stream and the fusiform gyrus (see Fig. 3D and Table 4).

Rhythms in the gamma range (>30 Hz) were significantly also reduced in N3, but mostly in prefrontal cortex (particularly around orbito-frontal cortex) (see Fig. 3E,F). Sigma activity in the range of 12–15 Hz showed again a peak in frontal and centro-parietal brain regions, particularly in dorso-lateral prefrontal cortex and the superior-frontal cortex, together with some reduction in occipito-temporal area.

## Discussion

In the present study we recorded MEG-data from sleeping subjects in all non-REM sleep stages. Using individual brain anatomy, we computed power estimates at the cortical level in order to describe the distribution of commonly observed oscillatory patterns and neural synchrony in sleep. We provide a report of spectral power changes for the main frequency bands separately for each non-REM sleep stage lumping together periods from each stage (N1, N2, N3) without distinguishing periods of high activity from the quiet periods. We present our results in the figures showing changes in activity in the background anatomy after transforming the results to a common space. We also report on these changes for each sleep stage within specific brain areas defined by a novel, spatially fine-grained parcellation of the human brain, obtained by combining anatomical, functional and diffusion data^38^.

Before discussing specific results in the context of the existing literature, it is useful to highlight some general limitations of our study. First of all, as already pointed out in the “Results” section, we are using a Minimum Norm Estimate solution on a superficial grid modelling the pial surface. Even if the solution has been weighted for depth bias (see “Methods” section), it is known it has an intrinsic bias to superficial generators and might fail to correctly localize activity from deep structures, especially if the magnetic field is stemming from subcortical structures^43,44^. For this reason, even if defined in the atlas we are using, we do not emphasize results at more deep regions of interest. Moreover, differently from other studies^36,37^ in this first analysis we include both periods of low and high activity and graphoelements in the estimation of the cortical power. This might have an effect, especially in detecting power changes stemming from very focal sources or leading to a dominating effect of graphoelements in the corresponding frequency band. Further investigations on the current, possibly expanded, dataset is required to improve our results.

A few other limitations are related to the acquisition protocol. We want to stress that recording sleep data with MEG is not an easy task and inevitably poses problems: for example, subjects have to avoid movements, often they have difficulties falling asleep, they move etc… One of our experimental efforts was to collect a larger and more homogeneous dataset of whole night sleep in MEG with respect to what can be found in the current literature.

To better understand whether our current dataset could add to the field of MEG sleep research, we conducted a systematic meta-analysis of the 36 MEG sleep studies on healthy humans published so far. We focused our attention on information regarding whether and how the analysis was conducted at the source level and how/if head position was monitored during sleep. Moreover, we report which EEG setup was used in each study in order to accomplish sleep scoring. Also, we included the number of participants per study and the average sleep time and, where available, the best cases as well. Finally, the meta-analysis includes in which studies an acclimatization night has been accomplished. Results of the meta-analysis are summarized in Supplementary Table 1.

The meta-analysis highlighted that only four out of 36 studies exploit whole night recordings while other four included four participants having a full night sleep and three others a daytime nap. On average, a total of 2.8 hours is spent in sleep (best case 8 hours). The average sample size is 6.68 (best case 23). Furthermore, only 7 out of 36 studies included an acclimatization night. A total of 15 studies investigated sleep features at the cortical level using full brain imaging methods (MNE, beamformers, MFT etc..) while eight used an informed equivalent dipole modelling. In 17 contributions it was declared how head position has been monitored during sleep: in particular, in four cases continuous HPI has been used while in the other cases the position has been monitored at predefined intervals (3/15/20 minutes) or at the beginning and at the end of the session. Finally, 24 studies used devices with whole head coverage.

As outlined in the “Methods” and “Results” sections, we have, on average, 145 minutes of sleep per participant (best case 264,3 minutes) and a sample of nine participants. We did not include an acclimatization night and, as other 11 studies, we monitored head position not continuously. This led to additional limitations that have to be considered in interpreting results. First of all, even if we compensate for head movement within each 20 minutes block by computing session dependent MNE solutions (see “Methods”), residual small head movements might have masked focal sources of power change, especially in the gamma range. Moreover, the sample size is small if compared to the standard in cognitive neuroscience, but still in line, and above the average, for MEG sleep studies, especially if compared to the sample size of studies addressing power spectral changes at the cortical level during sleep^34–37^. Within this framework, we think that our experimental dataset is at least in line with previous literature on sleep in MEG and that our results, despite not free from limitations, can add relevant information to the field.

As regards specific results, in sleep stage N1 we observed significant differences in comparison to the awake state in the alpha band, but not in the other frequency bands. The fact that we did not find any spectral difference in delta and theta bands between N1 and wakefulness, as Ioannides *et al*.^37^ found, may be due to the fact that both delta and theta oscillations are present also during wakefulness, which we used as a contrast against which we established all spectral changes. Indeed, it has been shown that theta band is most enhanced in frontal regions during wakefulness^45,46^. Also, delta band activity during sleep has previously been investigated following the hypothesis that it may have a homeostatic role during sleep^47–49^. In this regard, it has been shown that the amount of slow wave activity systematically increased with the amount of preceding wakefulness^47,49^ and that during the night there is a growing decrease in delta band power^50^. Also, the existence of a slow-wave rebound resulting from sleep deprivation has been demonstrated^51^. In our study though, participants were not sleep deprived and this may have contributed to the resulting absence of any difference between N1 and wakefulness in delta band. However, we have also to consider that the N1 stage has very much in common with wakefulness. Indeed, as indicated in the manual for sleep scoring, it is characterized by alpha being attenuated and substituted by lower amplitude activity for more than the 50% of the epoch. This makes the contrast between N1 and wakefulness less clean compared to other contrasts in which the sleep state is not a continuity of the awake state. Furthermore, N1 is a heterogeneous stage that combines alpha rhythms from wakefulness, alpha suppression and theta rhythms. One of the limitations of our approach is that we do not differentiate high/low activity periods and this might contribute in failing to detect slighter changes like those detected by Ioannides *et al*.^37^ in delta and theta band. In the alpha band the decrease in spectral power we observed in N1, relative to wakefulness, matches previous literature. It has previously been described that during wakefulness alpha band increases in occipital cortex when the eyes are closed as a result of activation suppression^52,53^. This suppression is relieved once the first sleep stage is reached. Also, in our data, the spectral amplitude for alpha is strongly evident on the occipital cortex in the awake state. As the N1 stage is entered, this activity in the alpha band is reduced, confirming previous findings according to which high-frequency band activity in general decreases when subjects enter light sleep stages. Limitations due to the reconstruction method and the head movement correction may have affected also our results in beta, low and high gamma bands in which we do not find differences as in Ioannides *et al*.^36^, especially in the case of low and high gamma bands, typically characterized by focal sources.

In N2 we saw an increase and a cortical spread of the low-frequency range (slow wave, delta, and theta). Delta oscillations are not restricted to visual areas in occipital cortex, but also strongly present in temporal and frontal cortex. The theta band activity spreads into temporal and frontal cortex, showing a prominent focus in frontal and occipital cortex and early visual areas. This is in line with previous findings suggesting a general increase of low frequencies as the N2 stage is entered^9,36,37,54^. In N2 we also observed an increase in slow wave oscillations (0.2–1.2 Hz), which is in line with previous reports according to which slow wave oscillations are typical features of deeper sleep stages^55^. Murphy and colleagues^56^ used high-density EEG source modeling to show that individual spontaneous slow waves have distinct cortical origins, and propagate uniquely across the cortex, involving unique subsets of cortical structures.

For frequencies in the beta range, previous literature suggested that beta band activity continuously decreases from light sleep to deep sleep stages^57^. From these previous reports, we expected to find an increasing gradient towards negative values (awake state) in the contrast for the beta frequency band, as participants proceeded into the deeper sleep stages N2 and N3. This was the case. We observed stronger beta oscillations in the awake state compared to N2 and N3 sleep stages. In particular, in N2 beta band decreased especially at the level of occipito-temporal regions, with a smaller decrease also at the level of frontal regions.

As for low gamma (30–60 Hz) and high gamma (60–120 Hz), in the contrast between N2 and wakefulness, both frequencies show a decrease in N2 mostly on frontal regions. As before, it has to be taken into account, that other focal changes might be masked because of limitations of our experimental setup.

With respect to the sigma frequency spectrum, which consists of waxing-and-waning 12–15 Hz oscillations^58,59^, our data show stronger sigma activity in N2 as compared to the awake state in frontal and centro-parietal cortex, consistently with the reported topographies in previous EEG studies^60,61^. In this regard, a previous MEG experiment by Manshanden and colleagues^34^ already showed that the major source of MEG spindles activity is situated within the centro-parietal region and the posterior portion of the frontal lobes. Dehghani *et al*.^16^ also showed in an EEG/MEG combined experiment that spindles frequency power was detectable at the level of centro-parietal areas and in midline of frontal cortex^62,63^. Ventouras and colleagues^64^ demonstrated that sleep spindles could be reliably extracted by reconstructing the EEG through back-projection of separate groups of independent components. The intracranial current sources related to the SCs were found to be spatially stable during the time evolution of the sleep spindles. Anderer and colleagues^65^ reported simultaneously active cortical spindle sources, which differed in frequency by approximately 2 Hz and were located in brain regions known to be critically involved in the processing of sensory input. DelFelice and colleagues^66^ finally obtained EEG signal from some subjects during daytime napping and could identify in about two thirds of the participants two to three generators of slow spindles activity in frontal lobes, with additional sources in parietal and limbic lobes.

The most drastic changes in the frequency spectrum compared to the awake state occurred in N3. Here we found a greater presence of delta waves prominently in fronto-temporal regions. Proceeding from N2 to N3, there was a clear increase in delta band activity with progression into deeper sleep stages (for a direct statistical comparison between sleep stages N2 and N3, see supplementary material Figs. S2–4). This is in line with previous literature^36,37,67,68^. The increase of delta over frontal regions was interpreted as an effect of an active inhibition of frontal lobes responsible for executive functions and task monitoring^37^.

Also, in the theta band we observed a gradual increase in power from N2 to N3 (for a direct statistical comparison between sleep stages N1, N2 and N3, see supplementary material Figs. S2–4). However, the amplitude in theta frequency was much more prominent in early visual cortex close to the occipital pole. This is still in line with previous EEG findings reporting a posterior dominance of activity during non-REM sleep in theta band^69^. In later sleep stages (e.g., N3) theta band activity spread also into dorsal and medial cortical areas but kept its peak in the occipital cortex.

In the N3 stage we observed a further increase in slow oscillatory patterns as compared to the awake state and all previous sleep stages (for a direct statistical comparison between sleep stages N1, N2 and N3, see supplementary material Figs. S2–4). In particular, regarding the slow oscillations, our data show a steady gradient of power increase in the amplitude spectra from lighter sleep to deep sleep. This is consistent with previous literature assessing a recurrent presence of slow wave activity during N3 sleep stage^70^. In particular, our data show a prominence of slow waves in N3 along the temporal cortex and in the inferior-frontal cortex. This is partially in line with previous EEG work showing that slow oscillations are recorded mostly in electrodes located anteriorly to Cz^70^.

The strong suppression of alpha band activity in N3 we see in visual cortex is consistent with previous literature showing that the alpha rhythm is strongest in amplitude when subjects are resting with their eyes closed^53,71^. Also visual cortex is strongly connected with certain subcortical structures, like the thalamus, which have been argued to be generators of inhibitory alpha rhythms in cortex during the awake state^72^.

Also, the beta frequency band is known to be stronger in wakefulness than in deep sleep especially at frontal and occipito-temporal regions, which is consistent with the peaks in our source-reconstructions of that signal. For example, Armitage^57^ showed that beta band systematically decreased from light to deep sleep. Here we confirmed this result by providing source estimations with peaks in the lateral and medial frontal cortex as well as in occipital-temporal cortex. Laufs and colleagues^73^ compared the modulation of the power in the beta band with the BOLD signal of fMRI resting state studies and found that the power in the beta frequency band is positively correlated with haemodynamic fluctuations in the posterior cingulate, precuneus and temporo-parietal and dorsomedial areas. These regions were also included in the default mode network by Greicius *et al*.^74^ suggesting that beta rhythms are a signature of cognitive operations in a conscious state, such as feedback and expectation signals as well as motor suppression.

With regard to the cortical gamma rhythms, our data show that its activity is stronger in wake than in deeper sleep stages, resulting in a negative activation in our source reconstructions. This is especially the case in medial prefrontal cortex. It has indeed been shown that the ventro-medial prefrontal cortex shows a high predominance of gamma band activity in the power spectra^12,75^.

As for the sigma frequency spectrum (12–15 Hz), our data show stronger activity in deep sleep (N3 as compared to the awake state) in frontal cortex. In addition to this signature our data also showed a decrease in occipital cortex in N3 compared to wakefulness, with a pronounced peak in early visual cortex. However, this activity is probably related to the partially overlapping alpha band, which is stronger in the awake state with eyes closed. In this regard, also Manshanden and colleagues^34^ detected alpha activity in occipital areas when they looked for spindle activity.

## Methods

### Participants

Twelve healthy subjects (4 women, mean age 25.8 +/− 2.6 years), all free of medication and with no history of psychiatric or neurological disease took part in the study, which was approved by the Human Research Ethics Committee of the University of Trento and conducted in accordance to the Declaration of Helsinki. Informed written consent was obtained prior to the recordings. Participants were asked to refrain from caffeine consumption for the day of the experiment and from alcohol in the 24 hours preceding it. There was no adaptation night inside the MEG scanner.

Before the experimental session, all subjects filled out the Pittsburgh Sleep Quality Index (PSQI)^76^ and the Epworth Sleepiness Scale (ESS)^77^ in order to screen their sleep habits and easiness to fall asleep. As for the PSQI, our subjects’ average score was 3.16. This is an indication that our subjects’ sleep habits and easiness to fall asleep were in line with our requirements, with a total PSQI score <5 indicating good general sleep quality and the absence of sleep problems. After the recording session, each subject filled out a questionnaire regarding how easy it was during the recording to fall asleep, and to what extent they felt restored after the sleep recording session on a scale from 1 (not at all) to 5 (very much). Here, the average scores were 4 and 3.5, respectively. The data of three participants had to be discarded from further data analysis because they could not fall asleep inside the scanner environment.

### Procedure and data acquisition

All sleep recordings took place during the participants’ sleep cycles (usually between 10 pm and 3 am). Before every session, five minutes of empty room were acquired to capture instrumental noise and compute noise covariance matrices. Participants were asked to lie in supine position in a Neuromag VectorView MEG scanner with 306 channels (204 first order planar gradiometers, 102 magnetometers, Elekta Inc., Helsinki, Finland) in a magnetically shielded room with two layers (AK3B, Vacuum Schmelze, Hanau, Germany). The MEG was set at a sampling rate of 1KHz, with online high-pass filter at 0.03 Hz and anti-aliasing filter at 330 Hz.

**Figure 4:**
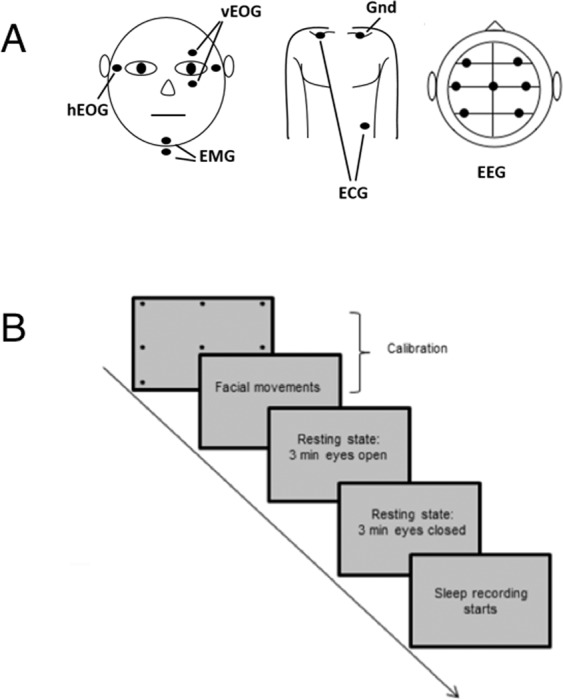
(**A**) montage of vertical and horizontal EOG, EMG, ECG, and EEG electrodes for the purpose of sleep classification and (**B**) sleep recording protocol with calibration and resting state recordings (with eyes open/closed) before the sleep session.

Together with MEG activity, standard polysomnographical (PSG) recordings were simultaneously acquired in order to classify sleep stages: using an MEG compatible 128 channels cap (Elekta Inc., Helsinki, Finland) 7 EEG electrodes were mounted (F3, F4, C3, CZ, C4, O1 and O2) in a 10–20 system, referenced to the left mastoid. Although the AASM manual^8^ only requires three derivations, we mounted additional, redundant EEG derivations as a backup. Vertical and horizontal electrooculography (EOG), chin electromyography (EMG) and electrocardiography (ECG) (right clavicle and abdomen with ground on the left clavicle, see Fig. 4A) were used as well. All electrode impedances, as measured before recording, were below 10 kΩ. At least one of the redundantly mounted EEG derivations for polysomnography was always less than 5 kΩ. Five Head Position Indicator (HPI) coils were used to determine the head position inside the MEG helmet. In order to co-register the MEG’s SQUID array with the participant’s individual head anatomy, three landmark positions at the left and right auricle points and nasion, and five head-position induction coils, which were attached to the forehead and the mastoids, were digitized along with a grid of at least 200 additional points, all evenly spread out over the subject’s head. For the digitization we used an electromagnetic position and orientation measuring system (Polhemus Inc., Vermont, USA). Landmarks and head-position induction coils were digitized twice to ensure that their spatial accuracy was less than 1 mm. At the beginning of each recording session, participants were asked to follow a short calibration procedure, in which they had to sequentially fixate on 9 black dots arranged in a 3 × 3 grid on a screen placed at a distance of 50 cm from their eyes, and then produce some facial movements (e.g. smiling and teeth grinding). The MEG/EEG recordings during these calibration procedures were later used for artifact identification and ICA decomposition. Following calibration, two resting states of 3 minutes each were recorded (see Fig. 4B). During the first resting state recording participants were asked to rest and fixate a cross, while in the second resting state recording they had to keep their eyes closed. During calibration and resting phase, lights were dim and head position was re-measured before each sequence. Participants were then instructed to try to fall asleep and lights were turned off. The continuous recording was intermitted and data was saved approximately every 20 minutes. At the beginning of each new recording period, a new head-position measurement was taken by electromagnetic induction in order to keep track of the head position inside the MEG helmet across the whole sleep session. In general, head movements did not exceed 1 cm. On average, interruptions between blocks lasted for 51 s (standard deviation 20 s; range 22–107 s).

### MEG data preprocessing

Each participant’s head position was checked across all subsequent recording blocks of the whole sleep session to assure that it remained stable across runs. Bad recording channels were excluded based on a visual inspection of the data (i.e., channels that were too noisy, flat or containing SQUID jumps). A maximum of 12 channels was excluded for each run. We applied Signal Source Separation (SSS)^78,79^ to the data, without using temporal extension and sensor data re-alignment; for each participant we set the origin of the multipole expansion spheres according to the digitized head shape. SSS decomposition matrices and sphere positions for each recording block has been saved for further empty-room recording preprocessing (see below).

On human data, residual muscle and sensor artifacts were visually detected and marked using Brainstorm software^80^. MEG data were resampled to 250 Hz and anti-aliasing low-pass filter set at 125 Hz. Welch’s PSD showed line peak frequency at 50 and 100 Hz, which were removed using notch filters. An extended Infomax Independent Component Analysis (ICA)^81^ was implemented to remove cardiac and eye-movement artifacts, separately on MEG gradiometers and magnetometers with the sampling frequency set at 250 Hz and high-pass filter at 0.1 Hz. The ICAs were implemented in a semiautomatic way: as for eye-movement artifacts, the independent components were calculated on each data run, concatenated with the subjects’ calibration runs in order to get the best possible separation. Manual inspection and removal of eye and cardiac related independent components were aided by thresholding linear correlation between the independent components and the HEOG, VEOG, and ECG channels, respectively.

The Minimum Norm Estimate Solution requires an empty-room recording for noise covariance matrix calculation. We further pre-processed the empty-room recording by applying the same temporal and spatial filters we used for human data. In particular, since here we are performing a different source reconstruction for each recording blocks and since each block is spatially filtered by means of SSS, we applied the SSS decomposition matrix to empty-room recording, so as to remove from the noise recording the same external components that have been removed from human data. This led to a specific preprocessed noise recording for each block of each subject.

### Sleep classification

Sleep stages were classified offline by two independent human experts inspecting PSG relevant data (EEG, EOG, ECG and chin EMG) on data epochs of 30 seconds. All sleep scoring followed the guidelines of the AASM manual^8^. For the purpose of sleep scoring, to-be-inspected EEG data were resampled to 250 Hz after a 125 Hz anti-alias filter and further filtered with 0.3 Hz low-pass filter and 35.0 Hz high-pass filter for visualization, while EMG and ECG data were low-pass filtered at 100 Hz. The two independent human scorers agreed on 98% of all epochs. Epochs of data for which the scorers did not agree were excluded from all subsequent analysis steps (2%).

### MRI acquisition and MRI-based source reconstruction of MEG signals

We obtained high-resolution T1-weighted anatomical scans of our subjects using a 4 T Bruker MedSpec Biospin MR scanner with an 8-channel birdcage head coil (MP-RAGE; 1 × 1 × 1 mm; FOV, 256 × 224; 176 slices; TR = 2700 ms; TE = 4.18 ms; inversion time (TI), 1020 ms; 7 degree flip angle). The anatomical scans were then 3D reconstructed using software^82,83^ and used in the 3D forward models of the MEG analyses.

We reconstructed neuronal sources using minimum-norm estimates (MNE)^84^, computing the forward solution with a multiple overlapping spheres model and using the correspondent preprocessed empty-room for noise covariance matrix calculation. The inverse kernel has been computed starting from the head position of each different recording block (measured every 20 minutes) thus compensating, at the source level, for head movements across blocks (see “Discussion” for limitations related to residual head movements within a recording block). All sensors, planar gradiometers and magnetometers, were used in the reconstruction and noise covariance matrices were regularized at 10%. Source space consisted of 15000 vertices and the orientation of the dipoles was fixed to the normal to the cortical surface. Moreover, depth weighting^85^ (order = 0.5; maximum weighting = 10) has been used, resulting in a partial attenuation of the superficial bias of the MNE solution (see “Discussion” for limitation of our approach in detecting deep sources). The 3D head-models were based on the individual segmentations of each participant’s MRI. Co-registration was based on the digitized fiducials and then refined to individual anatomy using the digitized additional points on the head’s surface. For one subject, MRI was not obtainable for technical reasons and a standard anatomy had to be used by transforming and warping the Freesurfer standard anatomy (MNI) to the subject’s head digitization data. All source-reconstruction steps and subsequent analysis were performed in Matlab with the Brainstorm toolbox^80^ and custom Matlab functions.

### Spectral analyses

For each subject, the amplitude spectral density (ASD) was computed separately for each stage from the previously computed sources using a Welch’s overlapping segments periodogram estimator (window length 4 s, corresponding to a 0.25 Hz spectral resolution). A spatial smoothing (Gaussian kernel, FWHM 2.0 mm) was applied to the cortical reconstructions. We investigated spectral features in the classical frequency bands. As previously mentioned, we averaged each subject’s spectra within delta (1–4 Hz), theta (4–8 Hz), beta (14–30 Hz), low gamma (30–60 Hz) and high gamma (60–100 Hz). As regards the alpha frequency band, usually broadly identified as 8–12 Hz, we detected the individual alpha frequency (IAF) for each subject and restricted the spectral analysis to that subject dependent frequency. We also included relevant frequency bands for the sleep, as slow waves (0.2–1.2 Hz) and sigma (12–15 Hz). Amplitude spectral densities, averaged over each subject’s sleep stage, were projected on the standard FreeSurfer template^86^ for group analysis. Finally, for each subject and sleep stage we reduced the correspondent amplitude spectral density to a set of 360 regions of interest (ROI) − 180 for each hemisphere – from a multi modal parcellation of the human brain^38^. The atlas we used has been defined by combining structural, diffusion, functional, and resting state MRI data from 210 healthy young adults. A single ASD for each parcel has been defined, as the maximal ASD across all the vertices within a given parcel of the atlas.

As a first group level analysis step we checked for inter-hemispheric differences. We used a parametric t-test for dependent samples, comparing each pair of ROIs between hemispheres, correcting for multiple comparisons using a false discovery rate *q* = 0.05. Since no significant differences between hemispheres were found for any of our ROIs, we averaged correspondent ROI values from the two hemispheres, thus reducing the effective number of parcels to 180. For each ROI we computed the difference of the subjects’ grand average, defining in this way contrasts between each specific sleep stage ASD (N1, N2, N3) and the ASD from awake resting state with eyes closed (Wake). A parametric dependent samples t-test was performed for each contrast for which we obtained cortical parcellated maps of significant differences of the means (p < 0.05, FDR corrected). The same procedure was used for internal contrasts between sleep stages (N2 vs N1; N3 vs N1; N3 vs N2).

## Conclusions

Our current data provides an overview of the cortical source reconstructions of changes in the oscillatory power spectra as human participants enter various sleep stages. Thanks to the spatial resolution of MEG, our analyses identifies systematic spectral changes in superficial regions of interest of a novel state of the art, spatially fine-grained parcellation of the human cortex. Despite the highlighted limitations, in our work, we managed to replicate findings from previous literature, for classical frequency bands, with a greater sample size compared to previous MEG sleep studies, spanning the whole spectrum and providing additional information regarding power spectral changes as subjects enter each of the NREM sleep stages.

## Supplementary information

Supplementary information.

## Data Availability

The datasets generated during and/or analyzed during the current study are available from the corresponding author on reasonable request.
