# Author Correction: Cortical source localization of sleep-stage specific oscillatory activity

**DOI:** 10.1038/s41598-020-65543-7

**Published:** 2020-05-20

**Authors:** Arianna Brancaccio, Davide Tabarelli, Marco Bigica, Daniel Baldauf

**Affiliations:** Center for Mind/Brain Sciences – CIMeC, University of Trento, Trento, Italy

Correction to: *Scientific Reports* 10.1038/s41598-020-63933-5, published online 24 April 2020

The Acknowledgements section in this Article is incomplete.

“We would like to thank Andrea Vitale for his help with code for data visualization. Also, we would like to thank the CIMeC research groups of Manuela Piazza, Marius Peelen, and Luca Turella for sharing previously acquired anatomical MRI scans for the cortical source reconstructions of some of our subjects.”

should read:

“We would like to thank Andrea Vitale for his help with code for data visualization. Also, we would like to thank the CIMeC research groups of Manuela Piazza, Marius Peelen, and Luca Turella for sharing previously acquired anatomical MRI scans for the cortical source reconstructions of some of our subjects. The study was supported by the Italian Ministry for Education, University, and Research (MIUR) project “Dipartimenti di eccellenza”.”

