# Supplementary material for: Cortical source localization of sleep-stage specific oscillatory activity
Source: Sci Rep. 2020 Apr 24;10:6976. doi: 10.1038/s41598-020-63933-5 (PMC7181624; doi:10.1038/s41598-020-63933-5)
Supplement: Supplementary file 1 — Supplementary information. [file 41598_2020_63933_MOESM1_ESM.pdf]

Cortical source localization of sleep-stage specific oscillatory activity

Supplementary information

Arianna Brancaccio*, Davide Tabarelli*, Marco Bigica, & Daniel Baldauf

Center for Mind/Brain Sciences – CIMeC
University of Trento, Italy

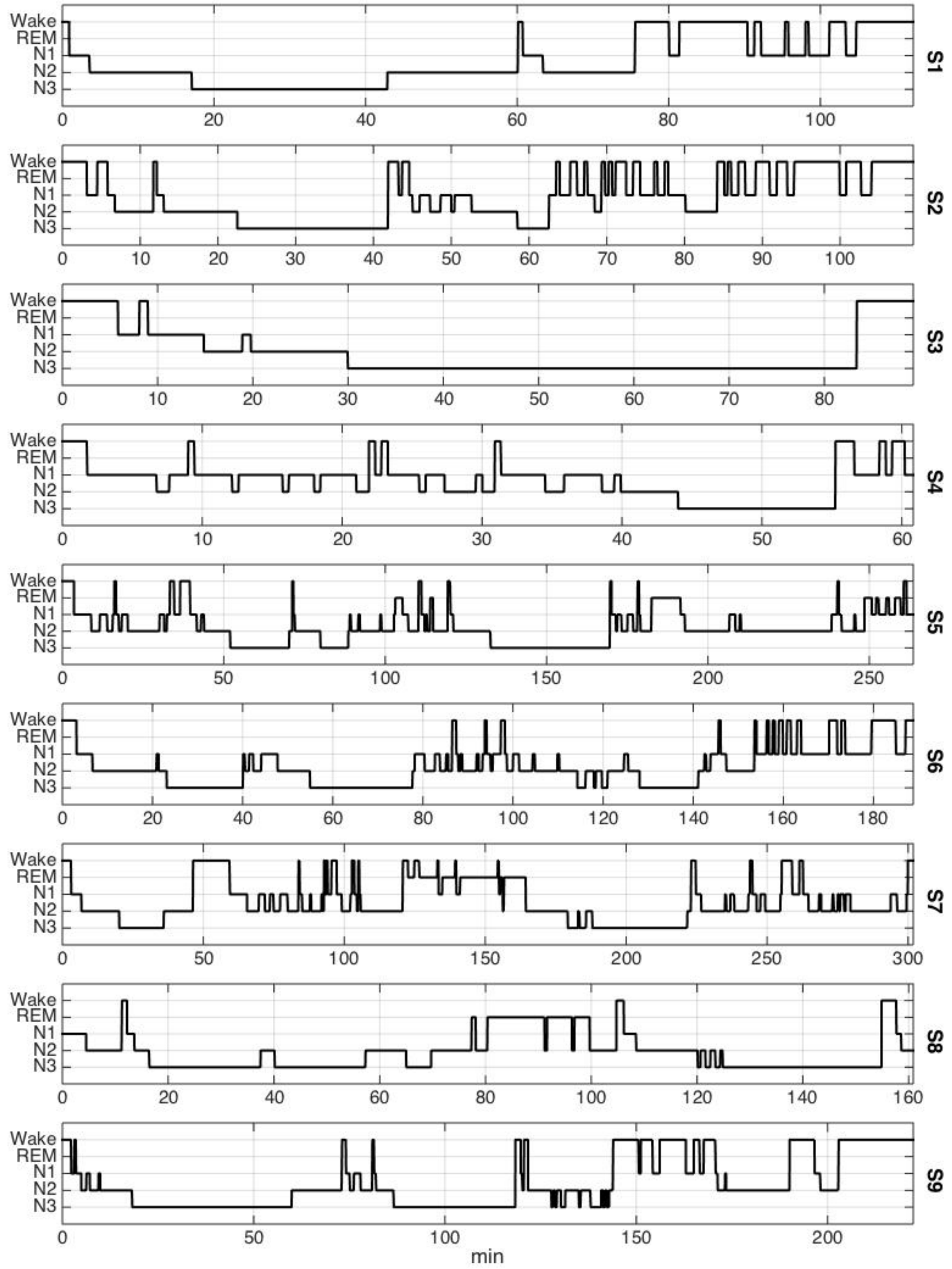

***Supp. Fig 1**:* Hypnograms for all subjects.

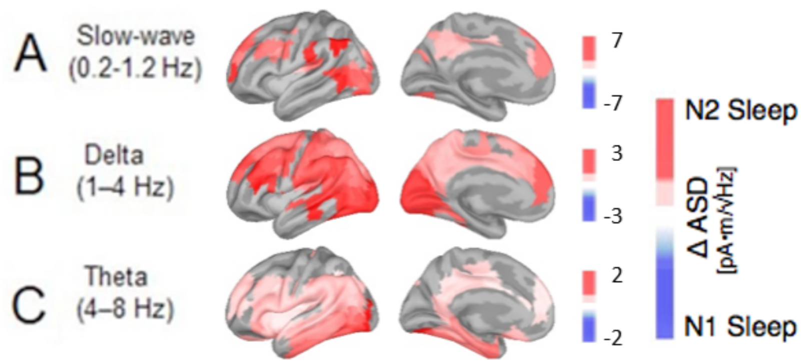

Supp. Fig. 2: Direct statistical comparisons between sleep stages N1 and N2 for the (A) slow wave (0.2-1.2Hz), (B) delta (1-4Hz), and (C) theta (4-8Hz) frequency range. Positive activation values (in red) refer to significantly stronger ASD in N2 than in N1. Negative activation values (in blue) refer to significantly stronger ASD in N1 than in N2. Non-significant differences are masked by $p<0.05$, FDR-corrected.

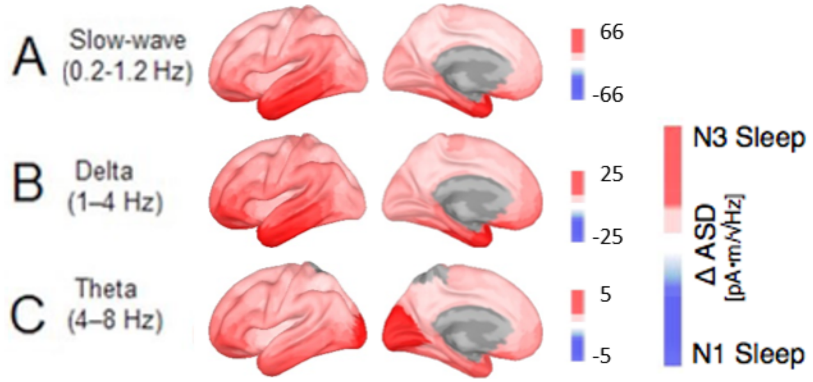

Supp. Fig. 3: Direct statistical comparisons between sleep stages N3 and N1 for the (A) slow wave (0.2-1.2Hz), (B) delta (1-4Hz), and (C) theta (4-8Hz) frequency range. Positive activation values (in red) refer to significantly stronger ASD in N3 than in N1. Negative activation values (in blue) refer to significantly stronger ASD in N1 than in N3. Non-significant differences are masked by $p<0.05$, FDR-corrected.

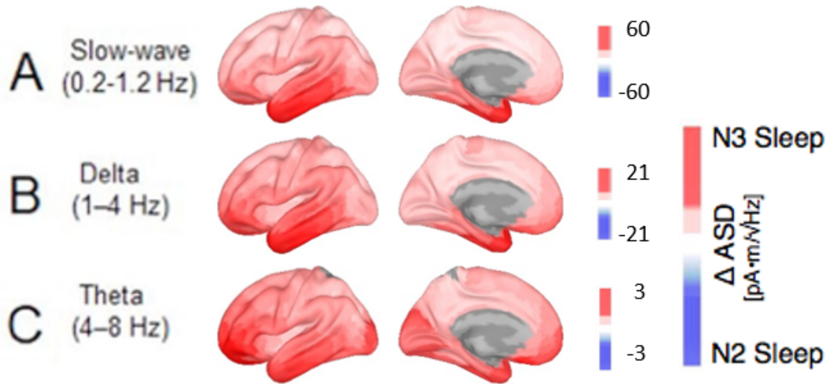

Supp. Fig. 4: Direct statistical comparisons between sleep stages N3 and N2 for the (A) slow wave (0.2-1.2Hz), (B) delta (1-4Hz), and (C) theta (4-8Hz) frequency range. Positive activation values (in red) refer to significantly stronger ASD in N3 than in N2. Negative activation values (in blue) refer to significantly stronger ASD in N3 than in N2. Non-significant differences are masked by $p<0.05$, FDR-corrected.

REFERENCE	YEAR	SUBJECTS	AVERAGE SLEEP TIME	MAXIMUM SLEEP TIME	HEAD POSITION MONITORING	EEG SETUP	ACCLIMATIZATION NIGHT	MEG HEAD COVERAGE	POSITION	SOURCE RECONSTRUCTION
Lu S.T. et al. [1]	1992	4	3 h	4 h		Fz, Cz, Oz	No	Partial (24 chan)	Supine	ECD
Llinas R. & Ribary U. [2]	1993	5	270 m	5 h		Fz-Cz, Cz-Pz	No	Partial (37 chan)	Supine	No
Ueno S. & Iramina K. [3]	1993					14 chan	No	Partial (7 chan)		ECD
Iramina K. & Ueno S. [4]	1994	1				14 chan	No	Partial (7 chan)		ECD
Iramina K. & Ueno S. [5]	1996					14 chan	No	Partial (7 chan)		ECD
Kitamura Y. et al. [6]	1996	6				Cz, Oz	No	Partial (74 chan)		ECD
Numminen J. et al. [7]	1996	6				Fz,Cz,Pz,Oz	No	Whole head	Upright	Yes
Yoshida H. et al. [8]	1996	1				14 chan	No	Partial (7 chan)		No
Naka D. et al. [9]	1999	10				Cz,Pz,Oz	No	Partial (74 chan)		No
Shih J.J. et al. [10]	2000	4	3 h			20 chan	No	Whole head		Yes
Simon N.R. et al. [11]	2000	3	33 m	50 m			No	Whole head	Upright	No
Manshanden I. et al. [12]	2002	4			3 HPI coils every 10 sec	64 chan	No	Whole head	Supine	ECD
Okusa T. & Kakigi R. [13]	2002	9	50 m	60 m		Cz, Pz, Oz	No	Partial (74 chan)	Supine	No
Ishii R. et al. [14]	2003	8	90 m	180 m		Fz, Pz, Cz, Oz	No	Whole head	Supine	Yes
Kakigi R. et al. [15]	2003	10/9/8 *	50 m	60 m	Video monitoring	Cz, Pz, Oz	No	Partial (74 chan)	Supine	ECD
Wang X. et al. [16]	2003	8				Cz,Pz,Oz	No	Partial (74 chan)	Supine	No
Ioannides A.A. et al. [17]	2004	3	6 h	405 m	HPI coils every 3 min	C3,C4	Yes	Whole head	Supine	Yes
Wang X. et al. [18]	2004	10				Cz,Pz,Oz	No	Partial (74 chan)	Supine	ECD
Corsi-Cabrera M. et al. [19]	2008	4				C3,C4	No	Whole head	Supine	No
Gumenyuk V. et al. [20]	2009	8	25 m	30 m	5 HPI coils every 15 min	C3, C4, O1, O2	No	Whole head	Supine	Yes
Ioannides A.A. et al. [21]	2009	4	6 h	405 m	HPI coils every 3 min	C3,C4	Yes	Whole head	Supine	Yes
Dehghani N. et al. [22]	2010	7	5.4 h **	8 h	5 HPI coils every 20 min	60 chan	No	Whole head	Supine	Yes
Dehghani N. et al. [23]	2010	7	5.4 h **	8 h	5 HPI coils every 20 min	60 chan	No	Whole head	Supine	No
Liu Z. et al. [24]	2010	7	40 m	45 mins	3 HPI coils begin/end of session		No	Whole head	Supine	No
Dehghani N. et al. [25]	2011	7	5.4 h **	8 h	5 HPI coils every 20 min	60 chan	No	Whole head	Supine	No
Dehghani N. et al. [26]	2011	7	5.4 h **	8 h	5 HPI coils every 20 min	60 chan	No	Whole head	Supine	No

Ayoub A. et al. [27]	2012	4	45 m	75 m		Cz,Fz	No	Whole head	Supine	No
Tamaki M. et al. [28]	2013	10	3 h		Continuous HPI	C3, C4, O1, O2, Fz, Cz, Pz	Yes	Whole head	Supine	Yes
Bang J.W. et al. [29]	2014	15	79 m		Continuous HPI	C3, C4, O1, O2	Yes	Whole head	Supine	Yes
Tamaki M. et al. [30]	2014	10	90 m ***	180 m	4 HPI coils begin of session	C3, C4, O1, O2, Fz, Cz, Pz	Yes	Whole head	Supine	Yes
Zerouali Y. et al. [31]	2014	5		90 m		56 chan	No	Whole head		Yes
Piantoni G. et al. [32]	2015	8	20 m				No	Whole head	Supine	Yes
Strauss M. et al. [33]	2015	13/23/9 *			Continuous HPI	60 ch	No	Whole head	Supine	Yes
Klinzing J.G. et al. [34]	2016	10	112 m	210 m	Continuous HPI	F3, FZ, F4, C3, Cz, C4	Yes	Whole head	Supine	Yes
Wen, H. & Liu Z. [35]	2016	5	40 m	45 m	3 HPI coils begin/end of session		No	Whole head	Supine	No
Ioannides A.A. et al. [36]	2017	4	6 h	405 m	HPI coils every 3 min	C3,C4	Yes	Whole head	Supine	Yes
**Our study**		**9**	**145**	**264,3**	**5 HPI coils every 20 min**	**C3, C4, Cz, F3, F4, O3, O4**	**No**	**Whole head**	**Supine**	**Yes**

Supplementary Table 1*: Results of the meta-analysis of the MEG sleep literature. Blank cells correspond to non-available data. In the last column, "ECD" stands for Equivalent Dipole Modeling, while "Yes" indicates more complete imaging methods (MNE, volumetric beamformers, etc …). Among the papers we cite, two of them do not provide the number of subjects in their samples, thus we did not include them in the count of the average number of participants per study. Two studies (*) perform three different experiments with different numbers of subjects. Four studies (**) collected whole night recordings of four participants and two-hour daytime naps on three participants. In one study (***) authors waked up participants after 90 or 180 minutes, analyzing only those first sleep cycle and then letting them sleep again until natural wake up, but without analyzing additional data.*

REFERENCES

1. Lu, S. T., Kajola, M., Joutsiniemi, S. L., Knuutila, J., & Hari, R. (1992). Generator sites of spontaneous MEG activity during sleep. Electroencephalography and clinical neurophysiology, 82(3), 182-196, (1992).

2. Llinas, R., & Ribary, U. Coherent 40-Hz oscillation characterizes dream state in humans. Proceedings of the National Academy of Sciences, 90(5), 2078-2081, (1993).

3. Ueno, S., & Iramina, K. Biomagnetic measurements and stimulation for imaging and estimation of the functional organization of the human brain. IEEE transactions on magnetics, 29(6), 3309-3314 (1993).

4. Iramina, K., & Ueno, S. Source localization of auditory evoked magnetic fields during waking and sleep stages. IEEE Transactions on Magnetics, 30(6), 4716-4718 (1994).

5. Iramina, K., & Ueno, S. Source estimation of spontaneous MEG activity and auditory evoked responses in normal subjects during sleep. Brain topography, 8(3), 297-301 (1996).

6. Kitamura, Y., Kakigi, R., Hoshiyama, M., Koyama, S., & Nakamura, A. Effects of sleep on somatosensory evoked responses in human: a magnetoencephalographic study. Cognitive brain research, 4(4), 275-279 (1996).

7. Numminen, J., Mäkelä, J. P., & Hari, R. Distributions and sources of magnetoencephalographic K-complexes. Electroencephalography and clinical neurophysiology, 99(6), 544-555 (1996).

8. Yoshida, H., Iramina, K., & Ueno, S. Source models of sleep spindles using MEG and EEG measurements. Brain topography, 8(3), 303-307 (1996).

9. Naka, D., Kakigi, R., Hoshiyama, M., Yamasaki, H., Okusa, T., & Koyama, S. Structure of the auditory evoked magnetic fields during sleep. Neuroscience, 93(2), 573-583 (1999).

10. Shih, J. J., Weisend, M. P., Davis, J. T., & Huang, M. Magnetoencephalographic characterization of sleep spindles in humans. Journal of Clinical Neurophysiology, 17(2), 224-231 (2000).

11. Simon, N. R., Manshanden, I., & da Silva, F. H. L. A MEG study of sleep. Brain research, 860(1-2), 64-76 (2000).

12. Manshanden, I., De Munck, J. C., Simon, N. R., & da Silva, F. H. L. (2002). Source localization of MEG sleep spindles and the relation to sources of alpha band rhythms. Clinical neurophysiology, 113(12), 1937-1947 (2002).

13. Okusa, T., & Kakigi, R. (2002). Structure of visual evoked magnetic field during sleep in humans. Neuroscience letters, 328(2), 113-116. (2002).

14. Ishii, R., Dziewas, R., Chau, W., Sörös, P., Okamoto, H., Gunji, A., & Pantev, C. Current source density distribution of sleep spindles in humans as found by synthetic aperture magnetometry. Neuroscience letters, 340(1), 25-28 (2003).

15. Kakigi, R., Naka, D., Okusa, T., Wang, X., Inui, K., Qiu, Y., ... & Watanabe, S. Sensory perception during sleep in humans: a magnetoencephalograhic study. Sleep medicine, 4(6), 493-507 (2003).

16. Wang, X., Inui, K., Qiu, Y., Hoshiyama, M., Tran, T. D., & Kakigi, R. Effects of sleep on pain-related somatosensory evoked potentials in humans. Neuroscience research, 45(1), 53-57 (2003).

17. Ioannides, A. A., Corsi-Cabrera, M., Fenwick, P. B., del Rio Portilla, Y., Laskaris, N. A., Khurshudyan, A., ... & Kostopoulos, G. K. MEG tomography of human cortex and brainstem activity in waking and REM sleep saccades. Cerebral cortex, 14(1), 56-72 (2004).

18. Wang, X., Inui, K., Qiu, Y., & Kakigi, R. Cortical responses to noxious stimuli during sleep. Neuroscience, 128(1), 177-186 (2004).

19. Corsi-Cabrera, M., Guevara, M. A., & del Río-Portilla, Y. Brain activity and temporal coupling related to eye movements during REM sleep: EEG and MEG results. Brain research, 1235, 82-91 (2008).

20. Gumenyuk, V., Roth, T., Moran, J. E., Jefferson, C., Bowyer, S. M., Tepley, N., & amp; Drake, C. L. Cortical locations of maximal spindle activity: magnetoencephalography (MEG) study. Journal of sleep research, 18(2), 245-253 (2009).

21. Ioannides, A. A., Liu, L., Poghosyan, V., & Kostopoulos, G. K. Using MEG to Understand the Progression of Light Sleep and the Emergence and functional Roles of Spindles and K-complexes. Frontiers in human neuroscience, 11, 313 (2017).

22. Dehghani, N., Cash, S. S., Chen, C. C., Hagler Jr, D. J., Huang, M., Dale, A. M., & amp; Halgren, E. (2010). Divergent cortical generators of MEG and EEG during human sleep spindles suggested by distributed source modeling. PloS One, 5(7), e11454 (2010).

23. Dehghani, N., Cash, S. S., Rossetti, A. O., Chen, C. C., & Halgren, E. Magnetoencephalography demonstrates multiple asynchronous generators during human sleep spindles. Journal of neurophysiology, 104(1), 179-188 (2010).

24. Liu, Z., Fukunaga, M., de Zwart, J. A., & Duyn, J. H. Large-scale spontaneous fluctuations and correlations in brain electrical activity observed with magnetoencephalography. Neuroimage, 51(1), 102-111 (2010).

25. Dehghani, N., Cash, S. S., & Halgren, E. Topographical frequency dynamics within EEG and MEG sleep spindles. Clinical Neurophysiology, 122(2), 229-235 (2011).

26. Dehghani, N., Cash, S. S., & Halgren, E. Emergence of synchronous EEG spindles from asynchronous MEG spindles. Human brain mapping, 32(12), 2217-2227 (2011).

27. Ayoub, A., Mölle, M., Preissl, H., & Born, J. Grouping of MEG gamma oscillations by EEG sleep spindles. Neuroimage, 59(2), 1491-1500 (2012).

28. Tamaki, M., Huang, T.-R., Yotsumoto, Y., Hamalainen, M., Lin, F.-H., Nanez, J. E., Watanabe, T., & Sasaki, Y. Enhanced Spontaneous Oscillations in the Supplementary Motor Area Are Associated with Sleep-Dependent Offline Learning of Finger-Tapping Motor-Sequence Task. Journal of Neuroscience, 33(34), 13894–13902 (2013).

29. Bang, J. W., Khalilzadeh, O., Hämäläinen, M., Watanabe, T., & Sasaki, Y. Location specific sleep spindle activity in the early visual areas and perceptual learning. Vision research, 99, 162-171 (2014).

30. Tamaki, M., Bang, J. W., Watanabe, T., & Sasaki, Y. The first-night effect suppresses the strength of slow-wave activity originating in the visual areas during sleep. Vision research, 99, 154-161 (2014).

31. Zerouali, Y., Lina, J. M., Sekerovic, Z., Godbout, J., Dube, J., Jolicoeur, P., & Carrier, J. A time-frequency analysis of the dynamics of cortical networks of sleep spindles from MEG-EEG recordings. Frontiers in neuroscience, 8, 310 (2014).

32. Piantoni, G., Van Der Werf, Y. D., Jensen, O., & Van Someren, E. J. Memory traces of long-range coordinated oscillations in the sleeping human brain. Human brain mapping, 36(1), 67-84 (2015).

33. Strauss, M., Sitt, J. D., King, J. R., Elbaz, M., Azizi, L., Buiatti, M., ... & Dehaene, S. (2015). Disruption of hierarchical predictive coding during sleep. Proceedings of the National Academy of Sciences, 112(11), E1353-E1362 (2015).

34. Klinzing, J. G., Mölle, M., Weber, F., Supp, G., Hipp, J. F., Engel, A. K., & Born, J. (2016). Spindle activity phase-locked to sleep slow oscillations. NeuroImage, 134, 607–616.

35. Wen, H., & Liu, Z. Separating fractal and oscillatory components in the power spectrum of neurophysiological signal. Brain topography, 29(1), 13-26 (2016).

36. Ioannides, A. A., Liu, L., Poghosyan, V., & Kostopoulos, G. K. Using MEG to Understand the Progression of Light Sleep and the Emergence and functional Roles of Spindles and K-complexes. Frontiers in human neuroscience, 11, 313 (2017).
